# Health information technology as a learning health system: Call for a national monitoring system

**DOI:** 10.1002/lrh2.10207

**Published:** 2019-11-20

**Authors:** Tiago K. Colicchio, Guilherme Del Fiol, James J. Cimino

**Affiliations:** ^1^ Informatics Institute University of Alabama at Birmingham Birmingham Alabama; ^2^ Department of Biomedical Informatics University of Utah Salt Lake City Utah

**Keywords:** adoption, electronic health records, learning health systems, medical informatics applications, outcome assessment

## Abstract

After over half a century of computer application development in medicine, the US health system has gone digital with an enthusiastic confidence for rapid improvements in care outcomes, especially those of quality of care, safety, and productivity. The bad news is that evidence for the justification of the hype around health information technology (HIT) is conflicting, and the expected benefits of a digital health system have not yet materialized. We propose a national system for monitoring HIT impact based on the paradigm of the learning health system (LHS): learning from practical experience through high‐quality, ongoing monitoring of care outcomes. Our proposal aims at leveraging current de facto standard research data repositories used to support large‐scale clinical studies by incorporating data needed for more robust HIT assessments and application of rigorous research designs that are now feasible on a large scale.

## 1 HEALTH INFORMATION TECHNOLOGY: EXPECTATIONS AND REALITY

After over half a century of computer application development in medicine, the US health system has gone digital. Most health care settings across the country have now adopted commercial electronic health record (EHR) systems with an enthusiastic confidence for rapid improvements in care outcomes, especially those of quality (eg, mortality and readmissions), safety (eg, medication errors and hospital‐acquired infections), and productivity (eg, care cost and provider efficiency). By 2009, EHRs had been adopted by 12% of US hospitals; today, adoption is above 90%.^1^ The bad news is that evidence for the justification of the hype around health information technology (HIT) is conflicting, and previous studies that have reported predominantly positive results are now being criticized for relying on weak research methods.^2^, ^3^ Evidence of the safety risks of HIT are starting to accumulate^4^ along with the need to finding definitive solutions, still resisted by current systems.^5^ Four years after going digital, the US health system is still the most expensive in the world and lags behind other developed countries in some important quality outcomes.^6^

HIT evaluations in which the EHR or its components were treated as interventions have served as the basis for the Meaningful Use (MU) program. However, most were single site studies, focused on home‐grown systems that have been discontinued and, in most cases, the studies provided putative, non‐generalizable outcomes.^7^ It is not surprising, then, that the expected benefits of a digitized health system have not yet materialized. However, as a result of nationwide adoption of a relatively small set of commercial EHRs, a large digital infrastructure is now available and can be leveraged to support more robust studies that were not feasible in the pre‐MU era. In this paper, we discuss the potential benefits of a national monitoring system of HIT impact to facilitate comparison of consensus outcomes across large, diverse sets of health care organizations implementing similar HIT tools “in the wild.” Hopefully, such a monitoring system would increase our understanding of the full impact of HIT interventions on care outcomes.

## 2 CURRENT STATE OF THE EVIDENCE PRODUCED BY PREVIOUS HIT EVALUATIONS

Care delivery in the United States is provided in fast‐paced, distributed care settings under constant adaptation to ever increasing medical knowledge. In such environments, implementation of a newly adopted EHR or the new version of an installed EHR—a complex process that can last several months—will inevitably add to the complexity of several aspects of care. These implementations tend to be ongoing sociotechnical processes, in which improved or new functionality is continuously implemented and system errors are constantly addressed. Such a cycle has no end since people continuously adapt to the technology and vice versa in order to optimize their work,^8^ which results in users constantly facing a learning curve that can last from months to years.^9^ Despite the complex and ever changing environment in which HIT is implemented, most HIT evaluations assess their interventions with simple short‐term pretest‐posttest designs.^10^ Such methods are not capable of detecting time‐sensitive effects^11^ that are common to HIT interventions.^12^ Due to the heterogeneity of systems used in the pre‐MU era and the challenges associated with data collection across multiple organizations, most studies are site‐specific and use a small set of nonconsensus measurements.^3^, ^10^ The use of agreed‐upon outcome measures that are shared among researchers is paramount to allow reproducibility of studies^13^ and the comparison of outcomes across studies of different sites by systematic reviews and meta‐analyses.^3^ Furthermore, lessons learned from other service sectors demonstrate that IT adoption rarely produces positive results if not accompanied by complementary factors or investments.^14^ Despite these lessons, HIT evaluations rarely consider potential context‐dependent factors that can affect their outcomes of interest.^10^

Finally, due to the high cost and complexity involved in EHR implementations and their ongoing maintenance, the definition of the implementation strategy, timeline, and settings to be implemented is naturally business‐driven decisions; in such cases, opportunities for randomization of intervention and control settings are limited, confirmatory studies are also difficult to conduct, and so a more practical approach to assess HIT impact on care outcomes is needed.

As large‐scale EHR implementations and their ongoing effects become ubiquitous, a robust national monitoring system can provide data needed to support future studies capable of comparing consensus outcomes across multiple settings using the same EHR system, generating a more agile, practice‐based learning process.

## 3 HIT IMPACT AND THE LEARNING HEALTH SYSTEM

The increasing evidence of medical errors and dependence on evidence‐based guidelines generated by artificially controlled research have contributed to establishing a new vision for improving US health care, referred to as the “learning health system” (LHS).^15^ The LHS proposes a paradigm shift in which medical knowledge would be primarily acquired from practical experience in naturalistic settings. By accessing large data sets and applying novel evaluations, known as “practice‐based evidence methods,” findings from traditional, expensive, and artificially controlled research methods such as randomized controlled trials can be augmented to develop more generalizable guidelines.

Understanding of the full impact of HIT interventions on care outcomes will require a paradigm analogous to the LHS: learning from practical experience through high‐quality, ongoing monitoring and pragmatic studies. Comprehensive quasi‐experimental methods for ongoing monitoring of HIT interventions^16^ and several pragmatic methods applicable to HIT research questions have been proposed recently.^17^ However, due to the constraints of large‐scale studies, they have mostly been applied to single‐site evaluations. By leveraging the digital infrastructure of the post‐MU era, a comprehensive database of consensus outcomes could be created, which could then be studied on a scale larger than ever before using methods such as interrupted time‐series designs with multiple control sites (for detection of ongoing effects) or pragmatic designs such as multisite clustered trials and stepped‐wedge designs (in which the EHR or an EHR's component is the intervention).

As necessary improvements are added to current EHRs and users adapt either to the multifunctional system or to isolated components added over time, continuous, near real‐time monitoring of HIT impact will be necessary to leverage future research. Care measures more likely to detect the impact of HIT interventions have been identified in the literature^3^ and combined with interviews and national surveys with subject‐matter experts.^18^ These measures assess care outcomes of quality, productivity, and safety and have already been tested in a large‐scale EHR implementation for identification of changes introduced immediately after the EHR go live or with long lasting effects.^12^, ^16^ That evaluation detected a significant impact attributable to the commercial EHR implementation in 40 (98%) measures monitored.^16^ An example includes a significant increase in the emergency department length of stay of four hospitals; the effect was observed only in the intervention sites and lasted from 10 to 15 months. A retrospective assessment of the same implementation revealed several internal and external context‐dependent factors that may have affected the longitudinally monitored outcomes, attesting to the fact that a full understanding of HIT impact will likely be reached only when such factors are considered.^19^ Several factors with the potential to affect care outcomes during HIT interventions can be quantitatively monitored with data readily available in electronic format such as provider‐patient ratio, changes to health insurance coverage, intentional changes to the volume patients, or seasonal variations. If such variables are collected from multiple organizations along with quality, safety and productivity outcomes, they can be analyzed as covariates or confounders to account for context‐dependent factors recognized as paramount for HIT appraisals,^17^ but rarely reported in the literature.^10^

Challenges related to technical aspects of data access and concerns about privacy and publicly available sensitive data will have to be managed for monitoring to occur beyond individual institutions. However, several measures that will likely be monitored may already be publicly available through benchmarking databases such the Centers for Medicare and Medicaid Services (CMS) Hospital Compare or the Healthcare Effectiveness Data and Information Set (HEDIS).^18^ These reporting systems include multiple quality and safety outcomes capable of detecting HIT impact^12^, ^16^ and could be combined with other measures from the National Quality Forum HIT safety measurements.^20^ To minimize the safety risks of HIT on a national level, an EHR oversight program^21^ and patient safety goals^22^ will need to be included explicitly as part of a national monitoring agenda. To capture data, one possible approach is to use de facto standard research data repositories like the Observational Health Data Sciences and Informatics (OHDSI)^23^ or the National Patient‐Centered Clinical Research Network (PCORnet).^24^ For example, PCORnet currently incorporates 13 clinical data research networks (CDRNs) that routinely collect care outcomes data (mostly quality and safety) from multiple organizations across the country to facilitate large‐scale clinical studies. To form the platform for a national monitoring system of HIT impact, such infrastructure could be leveraged to incorporate data that are not currently captured by CDRNs, but would be needed for monitoring HIT interventions, such as productivity‐related outcomes (eg, volume of visits, orders and hospitalizations), information about planned HIT interventions (eg, go live date of a new EHR component or a new EHR version), or data needed to monitor context‐dependent variables (eg, local insurance/market changes, seasonal variations, and provider‐patient ratio).^19^ The latter could be identified with guidance from implementation science frameworks such as the Consolidated Framework for Implementation Research (CFIR),^25^ which covers context domains such as inner and outer setting and individual characteristics that may influence the effect of HIT interventions. Another challenge is the business nature of most HIT interventions, which may hamper the possibility of randomization in the case of pragmatic studies. Since most integrated care delivery systems now use similar commercial EHRs, one approach would be to form groups of clients and organize a shared plan of implementation of new versions or system components, in order to orchestrate the randomization of these interventions.

Imagine a resource with data for several outcomes collected from multiple institutions on a regular basis or in real time depending on data availability. A dashboard built on top of such a resource would allow monitoring with statistical evaluation of longitudinal change patterns introduced by the implementation of a new EHR, a new EHR version, a new EHR component (eg, clinical decision support), outside applications, or system customization requests in a specific setting or group of settings spread across the nation. Each of these can be assessed as an individual intervention for identification of effects observed immediately after its introduction or effects observed over longer periods. For example, if modifications to a function used by infection prevention specialists decreases the specialists' efficiency, their capacity to investigate suspected infections may be hampered, resulting in an unexpected apparent decrease in the rate of hospital‐acquired infections. Studies using stepped‐wedge designs^26^ could be applied on subsequent implementations of the same product in different clients, confirming (or ruling out) a potential safety risk introduced by a new EHR version (infection rate could have decreased as a result of fewer cases investigated and not as a result of a real decrease in the number of infections). These effects could be shared with other institutions planning on implementing the same product or product component. Organizations not yet participating will pay their own way to join so that they can compare their systems with national experience and be informed about what to expect when they implement systems previously tested elsewhere, anticipating the need for organizational changes to prevent negative effects or to maximize positive ones. As mentioned above and demonstrated by previous HIT evaluations,^12^, ^16^ several measures that will likely be relevant to enable the proposed monitoring system are already captured on a regular basis by most care delivery systems across the country, and so the cost to develop the data feed to populate repositories such as PCORnet and OHDSI is likely to be manageable. Such a cost will surely be paid back many times over potentially improving provider satisfaction and productivity, as well as preventing quality and safety hazards.

Our approach will further LHS goals by allowing accumulation of evidence of HIT impact from practical experience to reach the larger medical and informatics communities in near real time, as opposed to lengthy site‐specific evaluations that, in addition to being less generalizable than what was desired, will be published long after their maximum usefulness, if they are published at all. The proposed monitoring system has the potential to lead us to a better understanding of the full impact of HIT on care outcomes and to avoid wasted time and money on another cycle of hundreds of studies that may not provide any valuable knowledge.

## CONFLICTS OF INTEREST

The authors have no competing interests to declare.
